# Diagnostic and Prognostic Utility of DNI and CRP in Patients with Dilated Cardiomyopathy

**DOI:** 10.3390/ijms27020871

**Published:** 2026-01-15

**Authors:** Nihat Söylemez, Özkan Karaca, Burak Toprak, Samet Yılmaz, Ahmet Turhan Kılıç

**Affiliations:** 1 Department of Cardiology, Mersin City Education and Research Hospital, Korukent Mah, 96015 Street Mersin Integrated Health Campus, 33240 Mersin, Turkey; drnihatsylmz@gmail.com (N.S.); md.ozkrc@gmail.com (Ö.K.); 2 Department of Cardiovascular Surgery, Mersin City Education and Research Hospital, Korukent Mah, 96015 Street Mersin Integrated Health Campus, 33240 Mersin, Turkey; ahmetturhank@gmail.com; 3 Department of Cardiology, Başkent University Adana Research Center, 01250 Adana, Turkey; sametyilmaz.dr@gmail.com

**Keywords:** dilated cardiomyopathy, inflammation, C-reactive protein, delta neutrophil index, mortality

## Abstract

Dilated cardiomyopathy is characterized by progressive left ventricular dilation and impaired systolic function, with inflammation recognized as a key contributor to disease onset and adverse outcomes. C-reactive protein reflects systemic biochemical inflammation, whereas Delta Neutrophil Index represents the circulating immature neutrophil fraction and provides a cellular dimension of inflammatory burden. The combined diagnostic and prognostic value of these two biomarkers in dilated cardiomyopathy has not been adequately explored. This retrospective study included one hundred and fifty patients with dilated cardiomyopathy and one hundred and fifty age-, diabetes-, and hypertension-matched controls. Demographic, laboratory, and echocardiographic measurements were analyzed. The diagnostic and prognostic performances of C-reactive protein, Delta Neutrophil Index, and their combined model were assessed using logistic regression, receiver operating characteristic curve analysis, reclassification metrics, calibration testing, and decision curve analysis. Additional analyses were performed for patients with left ventricular ejection fraction below twenty percent, and mortality predictors were examined within the dilated cardiomyopathy cohort. Both C-reactive protein and Delta Neutrophil Index levels were significantly higher in patients with dilated cardiomyopathy than in controls and were further elevated in those with severely reduced ejection fraction. Delta Neutrophil Index remained independently associated with severe left ventricular dysfunction (ejection fraction ≤ 20%) in multivariable analysis (odds ratio 2.51). Each biomarker showed an independent association with the presence of dilated cardiomyopathy, and their combined model achieved the highest diagnostic accuracy. In receiver operating characteristic analysis, the area under the curve was 0.895 for Delta Neutrophil Index, 0.691 for C-reactive protein, and increased to 0.920 for the combined model, with a sensitivity of 81.3% and specificity of 92.0%. Delta Neutrophil Index was independently associated with severe left ventricular dysfunction and mortality, while C-reactive protein, age, ejection fraction, urea, and sodium also contributed to mortality risk. Delta Neutrophil Index was independently associated with mortality (odds ratio 2.51), while C-reactive protein, age, ejection fraction, urea, and sodium also contributed to mortality risk. The combined model provided significant improvement in risk reclassification and demonstrated superior calibration and greater net clinical benefit across a wide range of decision thresholds. C-reactive protein and Delta Neutrophil Index offer complementary diagnostic and prognostic information in dilated cardiomyopathy. Their combined use enhances diagnostic discrimination, strengthens risk stratification, and improves identification of patients at high risk for severe ventricular dysfunction and mortality. Incorporation of these accessible biomarkers into clinical evaluation may support earlier recognition and more tailored management of high-risk individuals.

## 1. Introduction

Dilated cardiomyopathy (DCM) is a progressive myocardial disorder characterized by left ventricular dilatation and marked systolic dysfunction and represents one of the leading causes of heart failure in young and middle-aged populations [1]. The clinical course of the disease is highly heterogeneous; while some cases remain stable for years, others may deteriorate rapidly, resulting in recurrent decompensation episodes, advanced functional decline, and mortality [2]. Therefore, early recognition of DCM, accurate understanding of progressive remodeling processes, and timely identification of poor prognostic indicators constitute essential components of clinical management [1,2]. Beyond mechanical impairment, another dimension that has gained increasing importance in the pathogenesis of DCM is the persistent and multilayered effect of inflammation. Various experimental and clinical data have demonstrated that cyclical interactions among myocyte injury, oxidative stress, and immune system activation accelerate ventricular remodeling and significantly worsen the natural course of the disease [1].

Given the central role of chronic inflammation in DCM, inflammatory biomarkers have become major areas of interest in both diagnostic evaluation and prognostic stratification. C-reactive protein (CRP), one of the most frequently used markers, is a major acute-phase reactant synthesized in the liver and is considered a reflection of systemic inflammation across a broad spectrum of cardiovascular diseases [3]. In DCM, elevated CRP levels have been associated with increased cytokine release due to impaired myocardial cell integrity, tissue hypoxia, and accelerated fibrotic remodeling processes [4]. CRP elevation has been identified not only as an indicator of inflammatory activity but also as a marker linked to worsening ventricular geometry, declining ejection fraction, and increased mortality risk [3]. Therefore, CRP continues to serve as a valuable biomarker in understanding both the pathophysiological basis and the clinical outcomes of DCM.

Another inflammatory marker that has gained attention in recent years, the Delta Neutrophil Index (DNI), is a modern parameter that quantifies the immature neutrophil fraction using automated hematologic analysis. It was initially introduced for the early diagnosis of sepsis and severe inflammatory states [5]. However, growing evidence suggests that DNI may reflect not only acute infectious processes but also cardiovascular conditions associated with chronic inflammation, tissue injury, and immune dysregulation [6]. In DCM, it is hypothesized that persistent low-grade inflammation may impair neutrophil maturation in the bone marrow, leading to increased release of immature cells into circulation. Additionally, tissue hypoxia resulting from reduced cardiac output in advanced DCM may amplify systemic stress responses, resulting in elevated percentages of immature neutrophils. These biological mechanisms suggest that DNI may reflect not only inflammatory burden but also disease severity and cellular-level immune response dynamics [7]. Consequently, DNI has emerged as an increasingly attractive parameter for both diagnostic assessment and prognostic determination in DCM.

The fact that CRP and DNI represent different layers of inflammation suggests that their combined evaluation may provide a more comprehensive clinical perspective. While CRP reflects the biochemical component of systemic inflammatory response, DNI represents its cellular dimension; therefore, simultaneous use of these two markers may offer both a broad and in-depth view of inflammatory activity [8]. However, in the current literature, no comprehensive study has evaluated these two parameters together within the same cohort for DCM diagnosis, their association with an ejection fraction < 20%—a marker of poor prognosis—and their role in mortality prediction.

This study aims to investigate, through a large dataset consisting of 150 DCM patients and 150 control individuals matched for age, diabetes, and hypertension, the diagnostic value of CRP and DNI individually and in combination, their association with EF < 20%, and their predictive value for mortality. By doing so, it aims to establish a comprehensive model that elucidates the relationship of the inflammatory axis with disease burden, clinical deterioration, and mortality in DCM.

## 2. Results

The analysis first compared baseline demographic, clinical, and laboratory characteristics between the DCM and control groups. Subsequently, inflammatory markers, including DNI and CRP, were evaluated across subgroups stratified by left ventricular systolic function. Diagnostic performance analyses were then conducted to assess the ability of these biomarkers to discriminate DCM from controls. Finally, within the DCM cohort, the associations of DNI and CRP with mortality were examined using univariate and multivariable models. Detailed findings for each step of the analysis are presented below.

The data in Table 1 shows that the ejection fraction is significantly lower in the DCM group compared with the control group. Urea and creatinine levels are higher in patients with DCM, while GFR values are lower. Sodium and potassium levels are significantly increased in the DCM group. CRP and HbA1c levels are elevated in the DCM population. Albumin and calcium levels are significantly lower in patients with DCM. Hemoglobin and hematocrit values are lower in the DCM group. The delta neutrophil index (IG%) is markedly higher in DCM patients. MPV values differ significantly between the groups, and monocyte counts are lower in the DCM cohort. NT-proBNP levels are substantially higher in patients with DCM compared with control subjects. Mortality is notably higher in the DCM group compared with the control group (Table 1).

The data in Table 2 shows that potassium levels are significantly higher in patients with EF ≤ 20% compared with those with EF > 20%. CRP values are also elevated in the EF ≤ 20% group. In addition, the delta neutrophil index (IG%) is significantly increased in the severe EF ≤ 20% cohort compared with patients who have EF > 20%. NT-proBNP levels are markedly higher in patients with EF ≤ 20% than in those with EF > 20%. (Table 2).

The data in Table 3 show that the delta neutrophil index was strongly associated with the diagnosis of dilated cardiomyopathy in logistic regression analysis. In Model 1, DNI demonstrated a significant association with DCM with an odds ratio of 42.66 (95% CI, 17.80–102.26; *p* < 0.001). In Model 2, C-reactive protein was also significantly associated with the presence of DCM, with an odds ratio of 1.11 (95% CI, 1.07–1.15; *p* < 0.001). In the combined model including both biomarkers, DNI remained significantly associated with DCM (OR 44.86, 95% CI, 18.16–110.77; *p* < 0.001), and CRP also retained statistical significance (OR 1.12, 95% CI, 1.06–1.17; *p* < 0.001). The combined model demonstrated the highest explanatory power, with a pseudo R^2^ value of 0.502, and the likelihood ratio test was statistically significant (*p* < 0.001).

In Model 4, NT-proBNP was independently associated with the diagnosis of DCM, with an odds ratio of 1.55 per 1000 pg/mL increase (95% CI, 1.32–1.83; *p* < 0.001). In the fully combined model incorporating DNI, CRP, and NT-proBNP (Model 5), all three biomarkers remained significantly associated with DCM, with odds ratios of 20.0 (95% CI, 8.0–50.0; *p* < 0.001) for DNI, 1.07 (95% CI, 1.03–1.12; *p* < 0.001) for CRP, and 1.30 per 1000 pg/mL (95% CI, 1.12–1.52; *p* < 0.001) for NT-proBNP.

Receiver operating characteristic curve analysis demonstrated significant diagnostic performance for all models. The area under the curve was 0.895 for DNI with an optimal cut-off value of 0.90, yielding a sensitivity of 74.7% and a specificity of 92.7%. For CRP, the AUC was 0.691 with a cut-off value of 9.88 mg/L, corresponding to a sensitivity of 52.0% and a specificity of 83.3%. NT-proBNP also demonstrated strong diagnostic performance, with an AUC of 0.905 and an optimal cut-off value of 1000 pg/mL, yielding a sensitivity of 78.0% and a specificity of 90.0%. The combined DNI and CRP model achieved an AUC of 0.920. The fully combined DNI, CRP, and NT-proBNP model achieved the highest diagnostic accuracy, with an AUC of 0.945. At a predicted probability cut-off of 0.63, this model showed a sensitivity of 86.0% and a specificity of 93.0% (Table 3).

The data in Table 4 demonstrate significant associations between selected clinical and laboratory parameters, severe left ventricular systolic dysfunction, and mortality in patients with dilated cardiomyopathy. Potassium was significantly associated with an ejection fraction ≤ 20% (OR 3.01, 95% CI 1.18–7.63; *p* = 0.021). C-reactive protein was significantly associated with both severe systolic dysfunction (OR 1.05, 95% CI 1.01–1.11; *p* = 0.022) and mortality (OR 1.05, 95% CI 1.01–1.11; *p* = 0.022). The delta neutrophil index was significantly associated with EF ≤ 20% (OR 2.51, 95% CI 1.12–5.61; *p* = 0.026) and mortality (OR 2.51, 95% CI 1.18–5.35; *p* = 0.017). Age was significantly associated with increased mortality (OR 1.04, 95% CI 1.01–1.09; *p* = 0.013), while ejection fraction showed a significant inverse association with mortality (OR 0.92, 95% CI 0.88–0.96; *p* < 0.001). Urea levels were positively associated with mortality (OR 1.03, 95% CI 1.00–1.06; *p* = 0.042), whereas sodium levels demonstrated a significant inverse association with mortality (OR 0.89, 95% CI 0.81–0.98; *p* = 0.018) (Table 4).

The data in Table 5 demonstrate significant correlations between inflammatory markers and left ventricular systolic function, as well as marked effect size differences between patients with dilated cardiomyopathy and controls. Pearson correlation analysis showed a significant positive correlation between DNI and CRP (r = 0.27) and significant inverse correlations between DNI and ejection fraction (r = −0.60) and between CRP and ejection fraction (r = −0.23). Standardized mean difference analysis revealed large effect sizes for ejection fraction (SMD −3.72), delta neutrophil index (SMD 2.21), potassium (SMD 1.27), albumin (SMD −1.28), and mean platelet volume (SMD −0.96). Moderate effect sizes were observed for C-reactive protein (SMD 0.64), HbA1c (SMD 0.62), sodium (SMD 0.50), calcium (SMD −0.61), and urea (SMD 0.57) (Table 5).

The data in Table 6 demonstrate that the addition of inflammatory biomarkers significantly improved model performance and clinical utility. Reclassification analysis showed a significant net reclassification improvement when DNI was added to CRP (NRI 0.41, *p* = 0.004) and when CRP was added to DNI (NRI 0.19, *p* = 0.031), with corresponding significant improvements in integrated discrimination. The greatest improvement was observed when both biomarkers were added to the clinical model, resulting in significant increases in both NRI (0.47, *p* < 0.001) and IDI (0.112, *p* < 0.001). Calibration analysis of the combined model demonstrated acceptable goodness of fit, with a Hosmer–Lemeshow *p*-value of 0.557, a Brier score of 0.083, a calibration intercept of −0.04, and a calibration slope of 1.03. Decision curve analysis showed positive net benefit values for the combined model at clinically relevant risk thresholds of 0.10, 0.30, and 0.50, with net benefit values of 0.142, 0.111, and 0.066, respectively.

**Figure 1:**
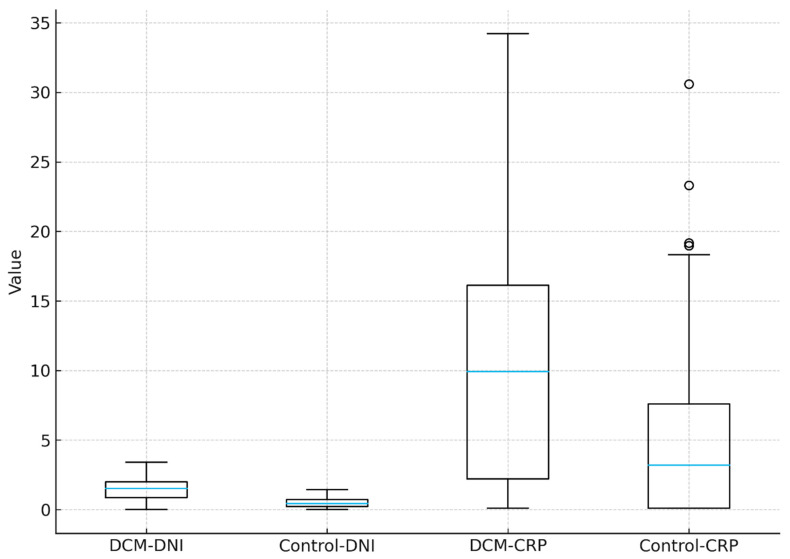
Distribution of delta neutrophil index (DNI) and C-reactive protein (CRP) levels in dilated cardiomyopathy (DCM) patients and control subjects. This boxplot illustrates the median values, interquartile ranges, whiskers, and outliers for DNI and CRP across DCM and control groups, providing a visual comparison of biomarker distributions between the two populations.

Figure 1 shows that DNI levels are higher in the DCM group than in the control group, with a wider distribution and elevated median values. CRP levels are also increased in patients with DCM, demonstrating broader variability and several high-value outliers, whereas the control group exhibits lower and more narrowly distributed values for both biomarkers (Figure 1).

**Figure 2:**
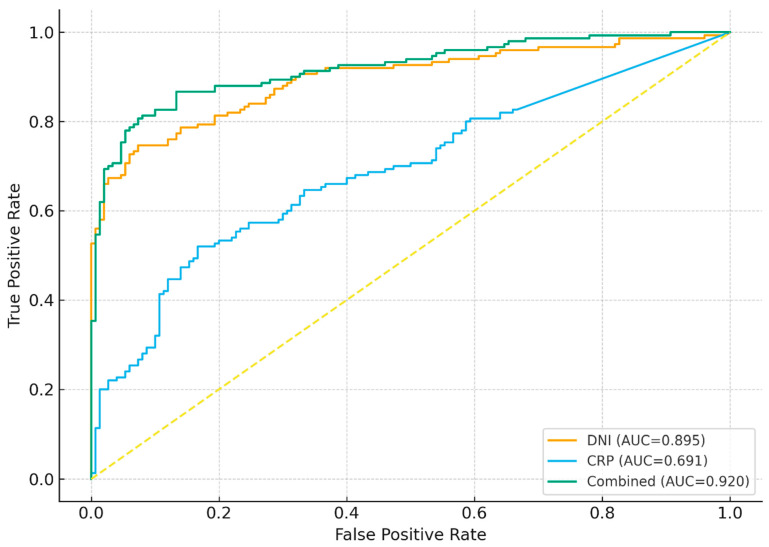
Receiver operating characteristic (ROC) curves for DNI, CRP, and the combined DNI + CRP model in the diagnosis of dilated cardiomyopathy. This figure shows the ROC curves for each diagnostic model, including corresponding area under the curve (AUC) values, and compares the discriminative performance of DNI, CRP, and their combined model.

Figure 2 shows that DNI demonstrates strong diagnostic performance with an AUC of 0.895, while CRP provides moderate discriminative ability with an AUC of 0.691. The combined DNI + CRP model yields the highest diagnostic accuracy, achieving an AUC of 0.920 (Figure 2).

**Figure 3:**
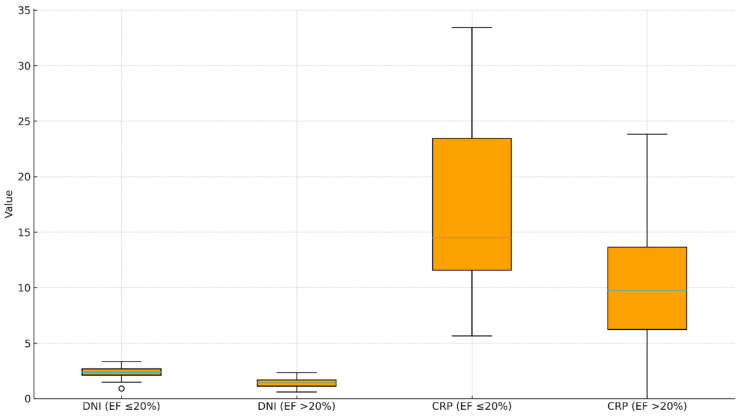
Distribution of delta neutrophil index (DNI) and C-reactive protein (CRP) levels in patients with EF ≤ 20% and EF > 20%. This boxplot depicts the median, interquartile range, whiskers, and outliers for DNI and CRP values stratified by severe (EF ≤ 20%) and mild–moderate (EF > 20%) left ventricular systolic dysfunction.

Figure 3 shows that DNI levels are higher in patients with EF ≤ 20% compared with those with EF > 20%, with a wider distribution and elevated median values in the severe EF group. CRP values are also increased in the EF ≤ 20% cohort, exhibiting broader variability and higher upper-range values, whereas patients with EF > 20% demonstrate lower and more narrowly distributed levels for both biomarkers (Figure 3).

**Figure 4:**
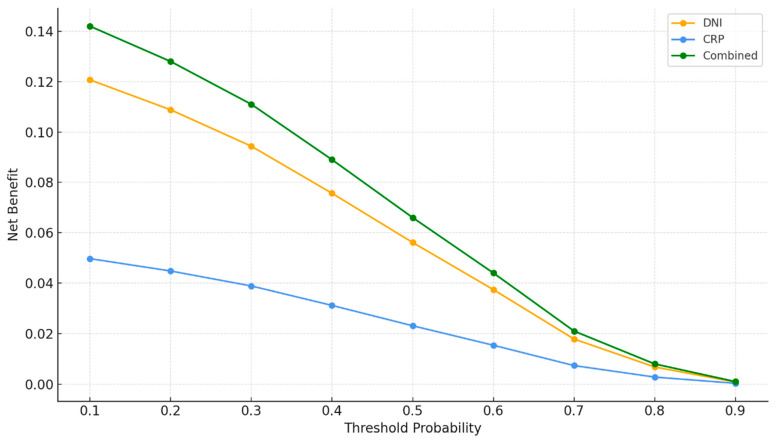
Decision curve analysis for delta neutrophil index (DNI), C-reactive protein (CRP), and the combined DNI + CRP model. This figure presents the net benefit curves across threshold probabilities ranging from 0.10 to 0.90, illustrating the clinical utility of DNI, CRP, and the combined model in the decision-making process for dilated cardiomyopathy (DCM) diagnosis.

Figure 4 shows that the combined model provides the highest net benefit across all threshold probabilities, outperforming both DNI and CRP individually. DNI demonstrates greater net benefit compared with CRP at each threshold, while CRP yields the lowest net benefit among the three models. The overall pattern indicates that incorporating both biomarkers offers superior clinical usefulness across a wide range of decision thresholds (Figure 4).

## 3. Discussion

In this study, we comprehensively evaluated the inflammatory biomarkers Delta Neutrophil Index and CRP levels in patients with dilated cardiomyopathy, both from diagnostic and prognostic perspectives. We demonstrated that both DNI and CRP levels were significantly higher in the DCM group compared with the control group, that these two markers were particularly elevated in the poor-prognosis subgroup with EF ≤ 20%, and that DNI was independently associated with mortality. From a diagnostic standpoint, DNI alone showed high discriminative power for DCM, while its combined use with CRP provided a clinically robust balance between sensitivity and specificity, with good model calibration performance and significant improvement in reclassification metrics (NRI, IDI). In logistic models constructed for mortality and reduced EF, adding DNI to clinical variables increased explanatory power, and the inflammatory axis approach, in which DNI and CRP were evaluated together, provided incremental value for both diagnosis and prognosis.

The central role of inflammation in the pathophysiology of DCM is a concept that has gained increasing support in recent years. However, it remains unclear whether inflammation represents a primary driving mechanism in idiopathic dilated cardiomyopathy or whether it predominantly reflects a secondary epiphenomenon accompanying myocardial injury and ventricular remodeling [2]. To date, the most definitive evidence supporting a causal inflammatory role in DCM derives from biopsy-proven, virus-negative inflammatory cardiomyopathy, in which targeted immunomodulatory strategies have shown potential benefit [9]. In the broader population of idiopathic DCM, inflammation is more likely to reflect a complex interplay between chronic myocardial stress, immune activation, and systemic neurohormonal dysregulation rather than a single primary trigger. The cyclical interaction between chronic myocardial injury, oxidative stress, and immune system activation has been identified as one of the key mechanisms that accelerate ventricular remodeling [1]. It has been shown that in conditions characterized by increased pro-inflammatory cytokines, altered neutrophil and monocyte responses, and accelerated fibrotic remodeling, left ventricular ejection fraction declines and clinical deterioration becomes more prominent [1,10]. In our study, the finding that DCM patients had higher DNI and CRP levels than a control group matched for demographic and conventional cardiovascular risk factors supports the notion that inflammatory burden is pronounced in this disease and that inflammation is not merely an accompanying feature but may represent one of the driving pathophysiologic forces. The choice of an ejection fraction threshold of ≤20% to define severe left ventricular systolic dysfunction requires specific justification. Although patients within intermediate ejection fraction ranges (such as 20–40% or 40–50%) represent a larger and clinically relevant population, these groups are inherently heterogeneous in terms of symptoms, remodeling patterns, and outcomes. In contrast, the European Society of Cardiology (ESC) describes patients with very low ejection fraction and refractory systolic dysfunction as representing an advanced-stage dilated cardiomyopathy phenotype, characterized by high mortality risk and progression toward end-stage heart failure. An ejection fraction ≤ 20% is commonly used in the literature as a pragmatic marker of this advanced disease state [9]. By focusing on this extreme phenotype, our aim was not merely to confirm its poor prognosis, which is already well established, but to investigate whether inflammatory biomarkers—particularly the Delta Neutrophil Index—provide additional discriminatory and prognostic information beyond what is evident from severe systolic impairment alone. This approach minimizes dilution of effect from intermediate phenotypes and aligns with our hypothesis that inflammatory burden becomes more pronounced and clinically informative in advanced-stage dilated cardiomyopathy.

CRP has long been used as an inexpensive and accessible biochemical indicator of systemic inflammation in many forms of cardiovascular disease [11,12]. Previous studies in heart failure and DCM populations have reported that elevated CRP levels are associated with reduced ejection fraction, higher NYHA functional class, and increased mortality [13]. From a pathophysiologic perspective, CRP elevation reflects underlying myocyte injury, IL-6–mediated hepatic acute-phase response, endothelial dysfunction, and fibrotic remodeling [14]. In our study, the observation that CRP levels were significantly higher in the DCM group compared with controls and that the increase in CRP was more pronounced in the EF ≤ 20% subgroup is consistent with these prior findings and suggests that CRP in DCM can be considered a marker not only of inflammatory burden but also of impaired ventricular function. However, although CRP reflects a strong acute-phase response, it does not directly capture the cellular components of inflammation or the flow of immature granulocytes; this highlights the need for more dynamic, cell-based parameters. In the present study, NT-proBNP levels were significantly higher in patients with dilated cardiomyopathy compared with control subjects and showed a marked increase in those with severe systolic dysfunction (EF ≤ 20%). Consistent with its established role as a marker of myocardial wall stress and neurohormonal activation, NT-proBNP demonstrated independent diagnostic value in logistic regression analysis. Moreover, the inclusion of NT-proBNP in multivariable models improved overall diagnostic performance, and the fully integrated model combining NT-proBNP with inflammatory biomarkers achieved the highest discriminative accuracy.

Importantly, these findings indicate that NT-proBNP and inflammatory biomarkers reflect complementary pathophysiological mechanisms in DCM. While NT-proBNP primarily captures hemodynamic burden and ventricular remodeling, biomarkers such as CRP and the Delta Neutrophil Index represent systemic and cellular inflammatory activity. The superior performance of the combined model suggests that advanced DCM is characterized by the coexistence of neurohormonal activation and persistent inflammation, rather than being driven by a single dominant pathway.

From a clinical perspective, the simultaneous assessment of NT-proBNP together with inflammatory markers may enable more refined risk stratification, particularly in patients with heterogeneous clinical presentation or borderline echocardiographic findings. In routine clinical practice, two-dimensional echocardiography combined with Doppler flow analysis remains the cornerstone of diagnostic evaluation in patients with suspected dilated cardiomyopathy and heart failure with reduced ejection fraction. Natriuretic peptides primarily reflect myocardial wall stress and volume overload, whereas inflammatory biomarkers provide complementary information regarding systemic and cellular immune activation. In patients presenting with dyspnea of uncertain cardiac or respiratory origin, suboptimal echocardiographic windows, or borderline functional findings, the combined assessment of DNI, CRP, and natriuretic peptides may contribute to a more comprehensive clinical interpretation and improve early risk recognition. This multidimensional biomarker approach may therefore offer incremental value beyond the use of natriuretic peptides or inflammatory markers alone in the evaluation of disease severity and prognosis in dilated cardiomyopathy. The clinical implications of risk reclassification based on inflammatory biomarkers should be interpreted as hypothesis-generating rather than directive at the current stage. Elevated DNI and CRP levels may identify a subgroup of patients with heightened inflammatory burden who could benefit from closer clinical follow-up, intensified optimization of guideline-directed medical therapy, or referral for advanced imaging modalities. Whether such patients should be considered for anti-inflammatory treatment strategies or endomyocardial biopsy remains uncertain and cannot be inferred from the present data. These questions represent important areas for future prospective studies aimed at defining the role of inflammation-guided management strategies in idiopathic dilated cardiomyopathy.

DNI represents the clinical translation of automated hematology analyzers’ ability to quantitatively measure the immature granulocyte fraction and was initially described for the early diagnosis of sepsis and severe infections [15]. Subsequent studies have demonstrated that DNI is closely related not only to infection but also to systemic inflammation, tissue stress response, and severe critical illness burden [5,6]. In the cardiovascular field, accumulating evidence suggests that DNI may have prognostic value in association with acute coronary syndromes, the inflammatory response after cardiopulmonary bypass, and ICU mortality [5,6,16]. Our study is important in that it shows DNI to be a meaningful biomarker from both diagnostic and prognostic standpoints in a chronic but exacerbation-prone cardiomyopathy such as DCM. The finding that DNI levels were significantly higher in the DCM group than in the control group, further increased in patients with EF ≤ 20%, and were independently associated with mortality in logistic regression analysis suggests that the immature neutrophil fraction may play a critical role even in the background of chronic inflammatory cardiomyopathy.

The positive correlation identified between DNI and CRP and the inverse correlations of both markers with ejection fraction in correlation analysis present a coherent biological picture supporting the concept that left ventricular function deteriorates as inflammatory burden increases. The particularly weak but significant correlation between DNI and EF suggests that this parameter may reflect not only the acute-phase response but also cellular response components associated with myocardial dysfunction. In recent years, speckle-tracking echocardiography (STE) has emerged as a valuable noninvasive imaging modality for the assessment of myocardial deformation and subclinical myocardial dysfunction in patients with dilated cardiomyopathy. Global longitudinal strain and related strain-derived parameters have been shown to correlate closely with the extent of myocardial fibrosis, which represents a key structural substrate underlying adverse ventricular remodeling and progressive systolic dysfunction in DCM. Importantly, myocardial fibrosis is increasingly recognized as an inflammation-driven process, mediated by chronic immune activation, cytokine signaling, and maladaptive extracellular matrix turnover. Previous studies have demonstrated that impaired strain parameters assessed by speckle-tracking techniques are associated with elevated inflammatory markers and worse clinical outcomes, supporting a mechanistic link between inflammation and myocardial structural damage [17,18]. In this context, the observed associations between elevated Delta Neutrophil Index, increased C-reactive protein levels, and reduced ejection fraction in the present study may reflect an underlying inflammatory–fibrotic continuum that could potentially be captured by advanced echocardiographic deformation imaging. Although speckle-tracking data were not available in our cohort, integrating inflammatory indices such as DNI and CRP with strain-based echocardiographic markers may offer a more comprehensive and noninvasive approach to phenotyping disease severity and progression in dilated cardiomyopathy. These findings strengthen the notion that an inflammatory axis approach, simultaneously evaluating both the biochemical (CRP) and cellular (DNI) faces of the myocardial remodeling process, may provide a more realistic assessment of disease burden in DCM.

From a diagnostic perspective, the ROC curve obtained for DNI in our study showed that this parameter alone has high discriminative ability in distinguishing DCM from the control population. CRP similarly demonstrated significant diagnostic performance; however, in the combined model including both markers, the AUC increased to 0.920, indicating a level of sensitivity–specificity balance that is highly satisfactory for clinical decision-making. The sensitivity of 81.3% and specificity of 92.0% achieved at the optimal cutoff suggest that the combined inflammatory model may provide a clinically meaningful balance between false negatives and false positives in DCM diagnosis, particularly as a complementary tool alongside conventional clinical and echocardiographic evaluation. Additionally, the good calibration of this combined model as evidenced by a nonsignificant Hosmer–Lemeshow test, low Brier score, and a calibration slope very close to 1 indicates not only high discriminative capacity but also that the predicted probabilities closely match observed outcomes. In clinical practice, the coexistence of strong ROC metrics and good calibration is critical for the reliable use of risk prediction models.

From the standpoint of reclassification analyses, our study showed that NRI and IDI values significantly increased in the model combining DNI and CRP compared with models using either marker alone. In particular, the finding that NRI reached 0.47 and IDI 0.112 in the extended model that added DNI and CRP to clinical variables indicates that this inflammatory axis approach confers a substantial advantage in reallocating patients into correct risk categories. This suggests that some patients previously classified as low or intermediate risk based solely on clinical parameters may actually belong to a higher-risk category when DNI and CRP levels are taken into account, thereby allowing potential clinical interventions to be implemented in a more targeted manner. The observation in decision curve analysis that the combined model provided the highest net benefit over a wide range of threshold probabilities further translates this finding into a clinical context, since this method quantifies not only statistical significance but also expected clinical benefit in real-world decision-making.

There is a large body of literature evaluating CRP as a prognostic biomarker in DCM and heart failure populations. Some studies have reported that high CRP levels are associated with reduced EF, higher NYHA class, and increased hospitalizations, and have suggested that CRP may be an independent predictor of mortality and major cardiac events [19,20]. Conversely, other investigations have emphasized that the prognostic power of CRP may be limited compared with other clinical scores or natriuretic peptides and that it may be insufficient when used alone [21]. In our study, CRP was found to be significant from both diagnostic and prognostic standpoints; however, it performed more strongly when evaluated together with DNI, and discriminative ability increased markedly in the combined model. This supports the notion that although CRP remains a valuable inflammatory biomarker in DCM, it may be inadequate to capture the full clinical heterogeneity when used alone, and that a multi-biomarker approach may partially compensate for this limitation.

A review of the DNI literature generally shows that elevated DNI levels are associated with increased mortality, greater disease burden, and prolonged ICU stay, and that DNI can provide incremental value beyond conventional inflammatory markers [5,6,16]. However, comprehensive studies evaluating DNI from both diagnostic and prognostic perspectives specifically in chronic, non-ischemic or ischemic dilated cardiomyopathy are quite limited. In this context, our study makes an important contribution to the existing literature by demonstrating that DNI levels are significantly higher in DCM patients than in controls, are further elevated in the EF ≤ 20% subgroup, and act as an independent predictor of mortality. These findings suggest that DNI may serve as a biomarker that links inflammatory burden and clinical outcomes not only in acute and critical settings but also in chronic cardiomyopathies.

Scores and models combining inflammatory markers are attracting increasing attention in the fields of heart failure and coronary artery disease, and various studies have shown that indices such as neutrophil-to-lymphocyte ratio (NLR), platelet-to-lymphocyte ratio (PLR), systemic inflammatory indices, and composite scores may be associated with mortality and rehospitalization [22]. However, a substantial proportion of these studies are based on cumulative leukocyte ratios and do not incorporate more sophisticated hematologic parameters such as the immature granulocyte fraction. In addition, most contemporary heart failure risk models rely heavily on natriuretic peptides, which were not available in our dataset; therefore, the incremental value of the DNI–CRP axis over natriuretic peptide–based strategies could not be formally quantified in the present study. In our study, the combined evaluation of a classical acute-phase reactant such as CRP with a dynamic parameter representing the immature neutrophil fraction such as DNI offers a unique approach that integrates both biochemical and cellular aspects of inflammation under a single framework. The strong performance of the combined model—reflected in high AUC, good calibration, significant NRI/IDI, and superior net benefit in decision curve analysis—provides concrete support for the complementary nature of these two biomarkers.

From the perspective of clinical translation, incorporating DNI and CRP into routine assessment in DCM patients may enable earlier and more precise identification of individuals at high risk for EF ≤ 20% and increased mortality. The fact that DNI can be obtained from a complete blood count without additional cost, and that CRP can be easily measured in virtually all levels of healthcare settings, enhances the practical feasibility of the proposed inflammatory axis approach. In DCM patients with high DNI and CRP levels—particularly when EF is borderline or mildly reduced—closer clinical follow-up, more aggressive optimization of medical therapy, and earlier consideration of advanced imaging or device therapies may provide meaningful benefits in personalized risk management.

### Limitations of the Study

Although the findings of this study provide substantial clinical and methodological insights, several limitations should be considered. First, the study has a single-center and retrospective design; this increases the potential for selection bias and limits the ability to establish causal relationships. Validation of these results through larger, multicenter, prospective studies is necessary. Additionally, dilated cardiomyopathy (DCM) represents a heterogeneous disease spectrum, and ischemic, non-ischemic, genetic, and inflammatory etiologies were evaluated together under the same classification. The absence of detailed subgroup analyses may obscure potential differences in the behavior of inflammatory biomarkers across distinct DCM subtypes. Moreover, detailed information on guideline-directed medical therapy (e.g., renin–angiotensin–aldosterone system inhibitors, beta-blockers, mineralocorticoid receptor antagonists, and SGLT-2 inhibitors) and on recent decompensation status was not systematically incorporated into the models, which may have resulted in residual confounding by treatment intensity and disease severity.

Second, DNI and CRP measurements were obtained at a single time point, making it impossible to assess temporal changes in these parameters or their relationship with longitudinal clinical outcomes. The dynamic course of inflammatory biomarkers may provide additional prognostic information regarding both DCM progression and treatment response; however, due to the absence of serial measurements, trend analyses could not be performed. Although NT-proBNP was included as a conventional cardiac biomarker, serial natriuretic peptide measurements were not available, precluding evaluation of temporal interactions between inflammatory activation and neurohormonal stress.

Third, although mortality data were reliably recorded, detailed classification of the causes of death was not possible. In DCM, death may be cardiac in origin or may result from concomitant infections or other systemic complications. The inability to analyze specific causes of death limits interpretation of the precise mechanisms linking inflammatory biomarkers to adverse outcomes.

Fourth, echocardiographic assessments were performed within a single center under routine clinical conditions. Although inter-observer variability and device-related differences were minimized, they cannot be entirely eliminated. Additionally, although EF < 20% was accepted as a threshold for advanced systolic dysfunction and poor prognosis, myocardial fibrosis and structural remodeling were not directly assessed using cardiac magnetic resonance imaging, which could have provided a more comprehensive evaluation of disease burden.

Fifth, the diagnostic analyses were based on a case–control framework with an approximately equal distribution of DCM patients and controls, which does not reflect the true prevalence of DCM in the general population. Consequently, the predicted probabilities derived from logistic regression models and the associated calibration and decision curve analyses primarily describe model behavior within this enriched sample rather than providing directly generalizable absolute risk estimates. In this context, the principal strength of the diagnostic models lies in their discriminative performance rather than population-level risk prediction.

Finally, although the integration of inflammatory biomarkers with conventional clinical parameters—including NT-proBNP—improved risk stratification and yielded favorable reclassification metrics, the models proposed in this study have not undergone external validation. Validation in independent cohorts would enhance generalizability and support clinical implementation. In addition, the number of outcome events, particularly for mortality, was modest relative to the number of covariates included in multivariable analyses. Therefore, some degree of model overfitting and optimism in performance estimates (AUC, NRI, IDI, and calibration metrics) cannot be excluded. No internal validation procedures, such as bootstrapping or cross-validation, were performed; thus, the reported model performance should be interpreted with appropriate caution.

Despite these limitations, the study provides important clinical insights through its comprehensive evaluation of inflammatory and neurohormonal pathways in DCM from both diagnostic and prognostic perspectives, highlighting the combined DNI–CRP approach—when interpreted alongside NT-proBNP—as a promising framework for future research.

## 4. Materials and Methods

### 4.1. Study Design

This study was designed as a retrospective observational investigation. The study was conducted in accordance with the Declaration of Helsinki and was approved by the Clinical Research Ethics Committee of Mersin City Education and Research Hospital (approval number: 2025/15; approval date: 20 October 2025). A total of 150 patients diagnosed with dilated cardiomyopathy (DCM) and evaluated between January 2022 and November 2025 in the Cardiology and Cardiovascular Surgery clinics of Mersin City Training and Research Hospital were included in the study group. To form the control group, 150 individuals who presented during the same period and were similar to DCM patients in terms of age, diabetes, and hypertension were randomly selected. Patients in the control group were required to have a normal left ventricular ejection fraction on transthoracic echocardiography and no history of heart failure, dilated cardiomyopathy, significant valvular heart disease, or documented coronary artery disease. Although the control group included patients with cardiovascular risk factors such as diabetes mellitus and hypertension, all individuals underwent systematic evaluation for myocardial ischemia. Patients with active or prior ischemic heart disease were excluded based on normal left ventricular ejection fraction, absence of ischemic changes on electrocardiography, negative cardiac troponin levels, and, when clinically indicated, coronary angiography demonstrating no significant coronary artery disease. Therefore, both the dilated cardiomyopathy cohort and the control group represented non-ischemic cardiac populations, allowing evaluation of inflammatory biomarkers independent of ischemic myocardial injury. Individuals with acute infection, chronic inflammatory or autoimmune disorders, active malignancy, advanced chronic kidney disease (stage ≥ 4), or recent major surgery (within the last three months) were excluded from both the DCM and control groups. All patients’ medical records were reviewed through the electronic hospital database, and files with missing data were excluded. Thus, a complete-case analysis approach was adopted, and no imputation procedures were applied. The proportion of excluded records due to missing key variables was limited (approximately 5%), but the possibility of selection bias related to this exclusion cannot be entirely ruled out. The diagnosis of DCM was confirmed based on echocardiographic detection of left ventricular dilatation and significant reduction in ejection fraction, along with the exclusion of coronary artery disease or other structural heart disorders. In accordance with the current European Society of Cardiology (ESC) guidelines, dilated cardiomyopathy was defined as the presence of left ventricular dilatation and systolic dysfunction (left ventricular ejection fraction < 45%) that could not be explained by abnormal loading conditions or coronary artery disease. Severe left ventricular systolic dysfunction was defined as an ejection fraction ≤ 20%, which is widely accepted as a marker of advanced disease stage and poor prognosis in patients with dilated cardiomyopathy [9]. In line with current recommendations, patients with significant coronary artery disease, primary valvular disease, or specific cardiomyopathies (e.g., hypertrophic, restrictive, infiltrative) were excluded; therefore, the study population predominantly reflected idiopathic or non-ischemic dilated cardiomyopathy. Etiological subtyping beyond this broad non-ischemic framework was not systematically performed, and all eligible patients were analyzed within a single DCM cohort.

### 4.2. Data Collection

Demographic data, comorbidities, echocardiographic measurements, and laboratory results of all individuals were obtained from the hospital information management system. C-reactive protein (CRP) and Delta Neutrophil Index (DNI) measured from blood samples constituted the primary biomarkers of the study. DNI values were obtained from routine complete blood counts performed using an automated hematology analyzer (Sysmex XN-1000, Sysmex Corporation, Kobe, Japan), which calculates the immature granulocyte fraction based on the difference between myeloperoxidase and nuclear lobularity channels. The reference range for DNI in our laboratory is 0.0–0.4%.

Serum CRP concentrations were measured using a high-sensitivity immunoturbidimetric assay (Roche Cobas c501, Roche Diagnostics, Mannheim, Germany) and expressed in mg/L. All analyses were performed in the same central laboratory, and internal quality control procedures were conducted according to the manufacturer’s and institutional standards. Additionally, biochemical and hematological parameters such as hemoglobin, leukocytes, neutrophils, lymphocytes, platelets, albumin, and potassium were recorded. Echocardiographic evaluation was performed in accordance with the recommendations of the American Society of Echocardiography, and left ventricular ejection fraction (EF) measurements were specifically used in prognostic analyses. Mortality data for all DCM patients were verified using hospital records and the national death notification system. The index date was defined as the time of the first qualifying echocardiographic evaluation and blood sampling, and all-cause mortality was ascertained up to November 2025, yielding a median follow-up duration of 30 months (interquartile range: 22–38 months). Because the exact timing of death was not uniformly available for all patients, mortality was modeled as a binary outcome rather than using time-to-event methods. Patients with EF < 20% were considered to have poor prognosis and were analyzed separately.

### 4.3. Data Analysis

#### 4.3.1. Statistical Analysis

Statistical analyses were performed using a multilayered and stepwise approach appropriate to the study objectives. The distribution characteristics of continuous variables were assessed visually (histograms, Q–Q plots) and using the Kolmogorov–Smirnov test. Normally distributed variables were expressed as mean ± standard deviation, whereas non-normally distributed variables were expressed as median (interquartile range). For comparisons between two groups, Student’s *t*-test was used for normally distributed continuous variables, and the Mann–Whitney U test was used for non-normal distributions. Categorical variables were presented as numbers and percentages, and differences between groups were tested using the chi-square test or Fisher’s exact chi-square test when expected cell counts were low. All *p* values were two-tailed, and *p* < 0.05 was considered statistically significant.

Baseline demographic, clinical, echocardiographic, and laboratory characteristics of the DCM and control groups were summarized in Table 1. This table reported both *p* values from classical hypothesis testing and standardized mean difference (SMD) values to assess group balance and effect size. This allowed quantitative evaluation of whether the randomly selected control group was clinically well balanced with the DCM group regarding key covariates such as age, sex, diabetes, and hypertension. An absolute SMD value below 0.10 was interpreted as good balance.

Among DCM patients, those with an ejection fraction (EF) ≤ 20% were classified as the “poor prognosis” group, whereas those with EF > 20% formed the comparison group; the results were presented in Table 2. Student’s *t*-test or Mann–Whitney U test was used according to variable distribution, and chi-square test was used for categorical variables. Statistically significant findings were highlighted.

To evaluate the diagnostic performance of CRP and DNI in diagnosing dilated cardiomyopathy, logistic regression models were constructed (Table 3). Model 1 included only DNI, Model 2 included only CRP, and Model 3 incorporated a combined model including both DNI and CRP. Odds ratios (OR) with corresponding 95% confidence intervals (CI) were calculated for all models, and *p*-values were derived using the Wald test. Receiver operating characteristic (ROC) curves were generated using predicted probabilities obtained from these models, and the area under the curve (AUC) values were calculated (Table 3). Ninety-five percent confidence intervals for AUCs were reported, and differences between AUCs were compared using the DeLong method. Optimal cut-off values in ROC analysis were determined using the Youden index (J = sensitivity + specificity − 1); sensitivity, specificity, positive likelihood ratio, and negative likelihood ratio were reported for each cut-off.

To identify factors associated with severe left ventricular systolic dysfunction (ejection fraction ≤ 20%), univariate logistic regression analyses were initially performed (Table 4). All clinical and laboratory variables were tested individually, and variables with *p* < 0.10 were considered candidates for inclusion in the multivariable model. A multivariable logistic regression model incorporating clinically relevant covariates together with DNI and CRP was subsequently constructed. A fixed, parsimonious model based on clinical relevance and prior literature—rather than a stepwise selection approach—was preferred. Results were expressed as ORs with 95% CIs. Continuous variables were analyzed in their continuous form and, when clinically meaningful, as categorized variables; linearity with the logit was graphically assessed.

To assess predictors of mortality, logistic regression analyses were performed in patients with dilated cardiomyopathy using a binary outcome (death versus survival) (Table 4). Univariate analyses first evaluated the association between individual clinical and laboratory parameters and mortality. Subsequently, a fully adjusted clinical model composed of established clinical variables was constructed, and expanded models incorporating DNI, CRP, and the combined DNI + CRP parameters were evaluated. Odds ratios, 95% confidence intervals, and *p*-values were reported for all models. Overall model explanatory power was assessed using pseudo R^2^ (Nagelkerke R^2^), and incremental improvements in model performance were interpreted accordingly.

To further explore the relationships among DNI, CRP, and left ventricular systolic function, Pearson correlation analysis was performed, and correlation coefficients (r) were calculated (Table 5). This analysis aimed to quantify the association between DNI and CRP, as well as the linear relationships between each biomarker and ejection fraction. Variables representing overlapping biological pathways and demonstrating high correlation were not included together in multivariable regression models in order to minimize multicollinearity.

To quantitatively evaluate the incremental diagnostic and prognostic value of biomarker-based models, net reclassification improvement (NRI) and integrated discrimination improvement (IDI) analyses were conducted (Table 6). Risk predictions derived from single-biomarker models or the clinical model were compared with predictions from models incorporating DNI, CRP, or both biomarkers. Risk probabilities were categorized into clinically meaningful strata to calculate categorical NRI, assessing correct reclassification in event and non-event groups. IDI was calculated based on differences in mean predicted probabilities between models, reflecting improvements in discriminatory capacity. Ninety-five percent confidence intervals and *p*-values were reported to evaluate the statistical significance of reclassification improvements.

Calibration of the combined diagnostic model (DNI + CRP) was assessed using the Hosmer–Lemeshow goodness-of-fit test and additional calibration metrics (Table 6). The Hosmer–Lemeshow test compared observed and predicted event rates across deciles, with *p*-values greater than 0.05 indicating acceptable calibration. The Brier score was calculated to quantify overall prediction error, with lower values indicating better model performance. Calibration intercept and slope were computed to assess systematic over- or underestimation of risk and proximity to the ideal model (intercept = 0, slope = 1).

Finally, decision curve analysis (DCA) was performed to evaluate the net clinical benefit of biomarker-based models across a range of clinically relevant threshold probabilities (Table 6). Net benefit values were calculated by balancing true-positive and false-positive classifications and were compared with “treat-all” and “treat-none” strategies. This analysis provided a quantitative assessment of the potential clinical utility of the combined model in decision-making across varying risk thresholds.

All analyses used a statistical significance level of α = 0.05, and *p* < 0.05 was considered statistically significant. Although multiple comparisons were performed consistent with the study hypotheses, no formal multiple testing correction was applied because the analyses were primarily exploratory. Therefore, *p* values should be interpreted in conjunction with effect sizes, confidence intervals, and biological plausibility rather than as definitive evidence of causal associations.

#### 4.3.2. Software

All statistical analyses were performed using IBM SPSS Statistics for Windows, Version 26.0 (IBM Corp., Armonk, NY, USA) and MedCalc Statistical Software, Version 20.218 (MedCalc Software Ltd., Ostend, Belgium). ROC analyses, decision curve analyses, and reclassification assessments were conducted using MedCalc. A *p* value < 0.05 was considered statistically significant.

## 5. Conclusions

This study demonstrates that the inflammatory biomarkers DNI and CRP provide valuable diagnostic and prognostic information in patients with dilated cardiomyopathy. The significantly elevated levels of both markers in the DCM group, their further increase in the EF ≤ 20% subgroup, and the independent association of DNI with mortality collectively support the central role of inflammation in both the development and clinical progression of the disease. In diagnostic modeling, the combined use of DNI and CRP outperformed single-marker approaches in terms of AUC, reclassification metrics, and calibration; the combined inflammatory axis approach offered a stronger and more clinically applicable framework. These findings suggest that a comprehensive assessment of inflammatory burden in DCM may contribute to improved risk stratification and earlier recognition of high-risk patients, although prospective studies are needed before the DNI–CRP axis can be incorporated into routine therapeutic decision-making. With its ease of integration into routine clinical practice, the DNI–CRP axis appears to be a promising candidate for a more prominent role in the future management of DCM.

## Figures and Tables

**Table 1:** Baseline demographic, clinical, echocardiographic, and laboratory characteristics of the study population (*n* = 300).

Parameter	DCM (n = 150)	Control (n = 150)	*p*-Value
Age (years)	53.10 ± 14.20	54.61 ± 6.84	0.218
Ejection fraction (%)	29.84 ± 9.23	55.51 ± 3.21	**<0.001**
Urea (mg/dL)	43.39 ± 21.31	33.55 ± 11.56	**<0.001**
Creatinine (mg/dL)	0.97 ± 0.65	0.83 ± 0.47	**0.033**
GFR (mL/min/1.73 m^2^)	91.98 ± 28.39	104.21 ± 26.88	**0.010**
Sodium (mmol/L)	138.71 ± 3.34	137.07 ± 3.27	**<0.001**
Potassium (mmol/L)	4.71 ± 0.51	4.14 ± 0.38	**<0.001**
ALT (U/L)	23.51 ± 14.89	22.54 ± 12.06	0.633
AST (U/L)	25.59 ± 12.66	23.68 ± 8.95	0.156
CRP (mg/L)	11.52 ± 18.64	2.68 ± 6.15	**<0.001**
HbA1c (%)	6.25 ± 1.49	5.49 ± 0.91	**<0.001**
LDL cholesterol (mg/dL)	126.22 ± 37.49	120.72 ± 36.18	0.180
Total cholesterol (mg/dL)	188.46 ± 43.96	187.16 ± 35.91	0.887
HDL cholesterol (mg/dL)	41.66 ± 11.29	43.42 ± 9.86	0.165
Albumin (g/dL)	3.86 ± 0.48	4.41 ± 0.37	**<0.001**
Magnesium (mg/dL)	2.11 ± 0.26	2.10 ± 0.24	0.773
Calcium (mg/dL)	8.91 ± 0.46	9.18 ± 0.43	**<0.001**
Hemoglobin (g/dL)	13.24 ± 1.98	14.00 ± 1.53	**0.005**
Hematocrit (%)	39.69 ± 5.96	41.67 ± 4.58	**0.009**
WBC (×10^3^/µL)	8.72 ± 3.93	8.29 ± 2.48	0.292
Platelets (×10^3^/µL)	239.82 ± 83.76	252.18 ± 77.77	0.172
Delta neutrophil index (IG%)	1.72 ± 0.83	0.35 ± 0.28	**<0.001**
MPV (fL)	9.35 ± 1.58	10.61 ± 1.00	**<0.001**
Monocytes (×10^3^/µL)	0.53 ± 0.19	0.64 ± 0.26	**<0.001**
Lymphocytes (×10^3^/µL)	2.27 ± 0.90	2.42 ± 0.74	0.135
NT-proBNP (pg/mL)	2890 ± 3120	210 ± 180	**<0.001**
Male sex, n (%)	97 (64.7%)	99 (66.0%)	0.903
Female sex, n (%)	53 (35.3%)	51 (34.0%)	0.903
Diabetes mellitus, n (%)	39 (26.0%)	39 (26.0%)	1.000
Hypertension, n (%)	63 (42.0%)	66 (44.0%)	0.817
Mortality, n (%)	38 (25.3%)	10 (6.7%)	**<0.001**

Data are presented as mean ± standard deviation or median (interquartile range), as appropriate. Normally distributed continuous variables were compared using Student’s *t*-test, while non-normally distributed variables (such as CRP and DNI) were analyzed using the Mann–Whitney U test. Categorical variables were compared using the Chi-square test. Statistically significant *p*-values (<0.05) are shown in bold. DCM = Dilated cardiomyopathy, GFR = Glomerular filtration rate, CRP = C-reactive protein, HbA1c = Glycated hemoglobin, LDL = Low-density lipoprotein, HDL = High-density lipoprotein, WBC = White blood cell count, MPV = Mean platelet volume, IG = Immature granulocyte.

**Table 2:** Comparison of clinical and laboratory parameters between EF ≤ 20% and EF > 20% groups in DCM patients (n = 150).

Parameter	EF ≤ 20% (Severe) (n = 33)	EF > 20% (Mild–Moderate) (n = 117)	*p*-Value
Age (years)	55.3 ± 15.84	52.4 ± 15.80	0.515
Sex, male (%)	21 (63.6%)	76 (65.0%)	1.000
Sex, female (%)	12 (36.4%)	41 (35.0%)	1.000
Diabetes mellitus (%)	8 (24.2%)	37 (31.6%)	0.547
Hypertension (%)	13 (39.4%)	66 (56.4%)	0.126
Urea (mg/dL)	38.32 ± 19.42	44.82 ± 21.68	0.123
Creatinine (mg/dL)	1.06 ± 0.76	0.95 ± 0.62	0.358
GFR (mL/min/1.73 m^2^)	89.61 ± 32.15	90.50 ± 28.04	0.876
Sodium (mmol/L)	139.18 ± 3.73	139.53 ± 3.05	0.582
Potassium (mmol/L)	4.92 ± 0.40	4.65 ± 0.45	**0.012**
ALT (U/L)	26.69 ± 14.85	29.90 ± 21.19	0.422
AST (U/L)	27.72 ± 15.84	29.47 ± 18.58	0.628
CRP (mg/L)	**15.20 ± 10.40**	**10.10 ± 7.90**	**0.018**
HbA1c (%)	5.89 ± 0.99	6.02 ± 0.70	0.401
LDL cholesterol (mg/dL)	140.10 ± 30.83	122.69 ± 30.74	0.794
Total cholesterol (mg/dL)	205.62 ± 69.39	183.86 ± 75.80	0.486
HDL cholesterol (mg/dL)	36.86 ± 9.44	43.37 ± 8.53	0.770
Albumin (g/dL)	3.60 ± 0.43	3.93 ± 0.41	0.672
Magnesium (mg/dL)	1.92 ± 0.13	1.93 ± 0.10	0.668
Calcium (mg/dL)	9.23 ± 0.57	9.11 ± 0.41	0.178
Hemoglobin (g/dL)	12.20 ± 1.81	13.55 ± 2.05	0.524
Hematocrit (%)	36.0 ± 8.19	40.5 ± 6.61	0.141
WBC (×10^3^/µL)	9.90 ± 2.56	8.30 ± 4.02	0.183
Platelets (×10^3^/µL)	195.00 ± 80.11	255.26 ± 71.94	0.851
Delta neutrophil index (IG%)	**2.35 ± 0.90**	**1.60 ± 0.70**	**0.004**
MPV (fL)	9.37 ± 1.31	9.34 ± 1.65	0.940
Monocytes (×10^3^/µL)	0.50 ± 0.20	0.54 ± 0.19	0.227
Neutrophils (×10^3^/µL)	5.35 ± 3.04	5.80 ± 3.56	0.508
Lymphocytes (×10^3^/µL)	2.36 ± 0.93	2.25 ± 0.89	0.516
NT-proBNP (pg/mL)	4820 ± 3680	2160 ± 2410	**<0.001**

Statistical tests applied include *p* value: Student’s *t*-test for continuous variables and the Chi-square test for categorical variables. In the table, statistically significant values are marked in bold. The *p*-value represents the level of statistical significance, where values < 0.05 are considered significant. GFR = Glomerular filtration rate, CRP = C-reactive protein, HbA1c = Glycated hemoglobin, LDL = Low-density lipoprotein, HDL = High-density lipoprotein, WBC = White blood cell count, MPV = Mean platelet volume, IG% = Delta neutrophil index.

**Table 3:** Diagnostic performance of DNI and CRP for identifying dilated cardiomyopathy (n = 300).

Section	Variable/Model	OR (95% CI)	*p*-Value	AUC	Cut-Off	Sensitivity (%)	Specificity (%)
A. Logistic Regression Analysis	Model 1	DNI (IG%)	42.66 (17.80–102.26)	<0.001	—	—	—
	Model 2	CRP (mg/L)	1.11 (1.07–1.15)	<0.001	—	—	—
	Model 3	DNI (IG%)	44.86 (18.16–110.77)	<0.001	—	—	—
		CRP (mg/L)	1.12 (1.06–1.17)	<0.001	—	—	—
		Pseudo R^2^	0.502	—	—	—	—
		Likelihood ratio *p*-value	<0.001	—	—	—	—
	Model 4	NT-proBNP (per 1000 pg/mL)	1.55 (1.32–1.83)	<0.001	—	—	—
	Model 5	DNI (IG%)	20.0 (8.0–50.0)	<0.001	—	—	—
		CRP (mg/L)	1.07 (1.03–1.12)	<0.001	—	—	—
		NT-proBNP (per 1000 pg/mL)	1.30 (1.12–1.52)	<0.001	—	—	—
B. ROC Curve Analysis	Single biomarker	DNI	—	—	0.895	0.90	74.7
	Single biomarker	CRP	—	—	0.691	9.88 mg/L	52.0
	Single biomarker	NT-proBNP	—	—	0.905	1000 pg/mL	78.0
	Combined model	DNI + CRP	—	—	0.920	0.59 (predicted probability)	81.3
	Combined model	DNI + CRP + NT-proBNP	—	—	0.945	0.63 (predicted probability)	86.0

Logistic regression analysis was used to evaluate the diagnostic association of DNI and CRP with dilated cardiomyopathy, and results are presented as odds ratios (OR) with 95% confidence intervals (CI). Model discrimination was assessed using receiver operating characteristic (ROC) curve analysis, with area under the curve (AUC), optimal cut-off values, sensitivity, and specificity reported. Higher AUC values indicate superior discriminative performance. DNI = Delta neutrophil index; CRP = C-reactive protein; IG% = Immature granulocyte percentage; OR = Odds ratio; CI = Confidence interval; AUC = Area under the curve.

**Table 4:** Predictors of severe systolic dysfunction (EF ≤ 20%) and mortality in patients with dilated cardiomyopathy (n = 150).

Variable	EF ≤ 20% OR (95% CI)	*p*-Value	Mortality OR (95% CI)	*p*-Value
Potassium (mmol/L)	3.01 (1.18–7.63)	0.021	—	—
CRP (mg/L)	1.05 (1.01–1.11)	0.022	1.05 (1.01–1.11)	0.022
DNI (IG%)	2.51 (1.12–5.61)	0.026	2.51 (1.18–5.35)	0.017
Age (years)	—	—	1.04 (1.01–1.09)	0.013
EF (%)	—	—	0.92 (0.88–0.96)	<0.001
Urea (mg/dL)	—	—	1.03 (1.00–1.06)	0.042
Sodium (mmol/L)	—	—	0.89 (0.81–0.98)	0.018

Multivariable logistic regression analyses were performed to identify independent predictors of severe left ventricular systolic dysfunction (ejection fraction ≤ 20%) and all-cause mortality in patients with dilated cardiomyopathy. For mortality, results from the fully adjusted clinical model (including age, ejection fraction, urea, sodium, and inflammatory biomarkers) are presented. Odds ratios (OR) are reported with corresponding 95% confidence intervals (CI). Statistically significant p-values (<0.05) are shown in bold. CRP = C-reactive protein; DNI = Delta neutrophil index; IG% = Immature granulocyte percentage; EF = Ejection fraction; CI = Confidence interval.

**Table 5:** Correlation and effect size analysis of inflammatory markers and cardiac function in dilated cardiomyopathy.

Section	Variable	DNI	CRP	EF	SMD
Correlation Analysis (n = 150)	DNI	—	0.27	−0.60	—
	CRP	0.27	—	−0.23	—
	EF	−0.60	−0.23	—	—
Effect Size Analysis (n = 300)	Age (years)	—	—	—	−0.14
	Ejection fraction (%)	—	—	—	−3.72
	Urea (mg/dL)	—	—	—	0.57
	Creatinine (mg/dL)	—	—	—	0.25
	GFR (mL/min/1.73 m^2^)	—	—	—	−0.44
	Sodium (mmol/L)	—	—	—	0.50
	Potassium (mmol/L)	—	—	—	1.27
	ALT (U/L)	—	—	—	0.07
	AST (U/L)	—	—	—	0.17
	CRP (mg/L)	—	—	—	0.64
	HbA1c (%)	—	—	—	0.62
	LDL cholesterol (mg/dL)	—	—	—	0.15
	Total cholesterol (mg/dL)	—	—	—	0.02
	HDL cholesterol (mg/dL)	—	—	—	−0.16
	Albumin (g/dL)	—	—	—	−1.28
	Magnesium (mg/dL)	—	—	—	0.04
	Calcium (mg/dL)	—	—	—	−0.61
	Hemoglobin (g/dL)	—	—	—	−0.43
	Hematocrit (%)	—	—	—	−0.37
	WBC (×10^3^/µL)	—	—	—	0.12
	Platelets (×10^3^/µL)	—	—	—	−0.16
	Delta neutrophil index (IG%)	—	—	—	2.21
	MPV (fL)	—	—	—	−0.96
	Monocytes (×10^3^/µL)	—	—	—	−0.48
	Lymphocytes (×10^3^/µL)	—	—	—	−0.17

Pearson correlation analysis was performed to evaluate linear relationships between delta neutrophil index, C-reactive protein, and ejection fraction, with correlation coefficients (r) reported. Standardized mean difference (SMD) analysis was used to quantify effect sizes of clinical and laboratory variables between dilated cardiomyopathy and control groups, independent of sample size. Absolute SMD values of 0.20–0.49 indicate small, 0.50–0.79 moderate, and ≥0.80 large effect sizes. Clinically relevant effect sizes are shown in bold. DNI = Delta neutrophil index; CRP = C-reactive protein; EF = Ejection fraction; GFR = Glomerular filtration rate; HDL = High-density lipoprotein; LDL = Low-density lipoprotein; WBC = White blood cell count; MPV = Mean platelet volume.

**Table 6:** Model performance and clinical utility of the combined diagnostic model (n = 300).

Section	Comparison/Metric	Value	*p*-Value
Reclassification Metrics (NRI/IDI)	CRP → + DNI (NRI)	0.41	0.004
	CRP → + DNI (IDI)	0.086	0.003
	DNI → + CRP (NRI)	0.19	0.031
	DNI → + CRP (IDI)	0.024	0.028
	Clinical Model → + DNI + CRP (NRI)	0.47	<0.001
	Clinical Model → + DNI + CRP (IDI)	0.112	<0.001
Calibration Statistics	Hosmer–Lemeshow χ^2^	6.82	—
	Hosmer–Lemeshow *p*-value	0.557	—
	Brier Score	0.083	—
	Calibration Intercept	−0.04	—
	Calibration Slope	1.03	—
Decision Curve Summary (Net Benefit)	Threshold 0.10	0.142	—
	Threshold 0.30	0.111	—
	Threshold 0.50	0.066	—

Net reclassification improvement (NRI) and integrated discrimination improvement (IDI) analyses were performed to assess the incremental diagnostic value of adding inflammatory biomarkers to baseline and clinical models. Calibration of the combined model (DNI + CRP) was evaluated using the Hosmer–Lemeshow goodness-of-fit test, Brier score, calibration intercept, and slope. Decision curve analysis (DCA) was used to estimate the net clinical benefit of the combined model across selected clinically relevant risk thresholds (0.10, 0.30, and 0.50). Statistically significant *p*-values (<0.05) are shown in bold. DNI = Delta neutrophil index; CRP = C-reactive protein.

## Data Availability

The data presented in this study are available on request from the corresponding author.

## Abbreviations

CRP	C-reactive protein
DCM	Dilated cardiomyopathy
DNI	Delta Neutrophil Index
EF	Ejection fraction
NRI	Net Reclassification Improvement
IDI	Integrated Discrimination Improvement
ROC	Receiver operating characteristic
AUC	Area under the curve
LV	Left ventricle/Left ventricular
GFR	Glomerular filtration rate
WBC	White blood cell count
MPV	Mean platelet volume
